# miR-219-5p targets CaMKIIγ to attenuate morphine tolerance in rats

**DOI:** 10.18632/oncotarget.15997

**Published:** 2017-03-08

**Authors:** Jian Wang, Wei Xu, Jiali Shao, Zhenghua He, Zhuofeng Ding, Jiangju Huang, Qulian Guo, Wangyuan Zou

**Affiliations:** ^1^ Department of Anesthesiology, Xiangya Hospital, Central South University, Changsha, Hunan 410008, China

**Keywords:** morphine tolerance, microRNA, CaMKIIγ, NMDA

## Abstract

Morphine tolerance is a clinical challenge in pain management. Emerging evidence suggests that microRNA (miRNA) plays a regulatory role in the development of morphine tolerance. miR-219-5p (miR-219) targets calmodulin-dependent protein kinase II γ (CaMKIIγ) to activate central pain sensitization via N-methyl-D-aspartate (NMDA) receptor. Therefore, we hypothesized that miR-219-5p attenuates morphine tolerance by targeting CaMKIIγ. We found that the expression of miR-219-5p was decreased significantly after chronic morphine treatment. Overexpression of miR-219-5p by lentivirus injection prevents the development of morphine tolerance. CaMKIIγ, the target gene of miR-219-5p was downregulated by overexpression of miR-219-5p both *in vivo* and *in vitro*. Furthermore, we found that lentiviral-mediated miR-219-5p decreased the expression of NMDA receptor subunit 1 (NR1), leading to attenuation of morphine tolerance. Overall, the data demonstrate that miR-219-5p plays a crucial role in alleviating morphine tolerance by inhibiting the CaMKII/NMDA receptor pathway. Overexpression of miR-219-5p may be a potential strategy to ameliorate morphine tolerance.

## INTRODUCTION

Morphine is commonly used to alleviate moderate-to-severe pain, especially cancer pain. Prolonged administration of morphine leads to morphine tolerance, which requires higher doses of morphine to produce the same analgesic effect [1]. The specific mechanism underlying morphine tolerance still remains unknown. Recent evidence suggests that post-translational regulation by miRNAs may mediate the development of morphine tolerance [2–4].

MicroRNAs (miRNAs) are small non-coding RNAs containing 18~22 nucleotides, which regulate gene expression at the translational level [5]. miRNAs repress mRNA expression or destabilize mRNA by binding to the 3′-untranslated region (UTR) of the target genes [6]. Currently, thousands of miRNAs have been identified in humans, and are involved in the pathophysiology of various diseases [7]. Evidence indicates that miRNA is expressed abundantly in nervous system and serves as an important epigenetic regulator of neurobiological activity, including neurogenesis, neuronal plasticity and pain perception [8–11]. Morphine tolerance is of growing interest in the study of miRNA-mediated cellular adaptation.

Several studies investigated the regulatory role of miRNA in the development of morphine tolerance. A few miRNAs attenuate morphine tolerance by regulating μ-opioid receptor (MOR) expression. For example, Let-7 binds to 3′-UTR of MOR to repress its expression [12]. miR-23b acts as a trans-acting factor, which interacts with the k box motif of 3′-UTR of MOR1 to suppress MOR translation efficiency [13]. In addition to let-7 and miR-23b, other miRNAs involved in morphine tolerance include miR-124, miR-190, miR-103 and miR-93-5p [14–17]. In a previous study, we demonstrated the deregulation of nine different miRNAs in rat spinal cord after chronic morphine injection, including let-7, miR-365 and miR-219-5p (miR-219) [4]. Studies have demonstrated that miR-219 regulates NMDA receptor-mediated neurobehavioral dysfunction and neuropathic pain by targeting calmodulin-dependent protein kinase II γ (CaMKIIγ) [18, 19].

Considering the importance of NMDA receptor in morphine tolerance [20], we hypothesized that miR-219-5p attenuates morphine tolerance by targeting CaMKIIγ in rats.

## RESULTS

### Downregulation of miR-219-5p expression in the spinal cord of morphine-tolerant rats

**Figure 1:**
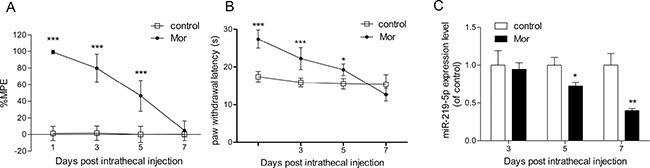
Chronic morphine treatment inhibits the expression of miR-219-5p (**A**) Tail-flick test of morphine tolerance. Tail-flick test was performed at 1, 3, 5, and 7 days before or 30 min after morphine or saline injection. Tail-flick latency was converted to %MPE (*n* = 6, ***P < 0.001, compared with control group, using two-way ANOVA followed by Bonferroni correction). (**B**) Thermal paw withdrawal latency test. Thermal paw withdrawal latency test was performed 30 min after morphine or saline injection. (*n* = 6, *P < 0.05, ***P < 0.001, compared with control group, using two-way ANOVA followed by Bonferroni correction). (**C**) Temporal changes in miR-219-5p expression after chronic morphine treatment. The expression of miR-219-5p in L4~L5 spinal cord was examined at 3, 5, and 7 days after morphine or saline injection by qRT-PCR. Values were normalized to those of U6 before comparison (*n* = 4, *P < 0.05, **P < 0.01, compared with control group, by Student's *t*-test). All the data were expressed as mean ± SD. Mor = morphine (10 μg/10 μL, twice daily) intrathecal injection for 7 days; control = saline (10 μL, twice daily) intrathecal injection for 7 days.

After 7 consecutive days of intrathecal administration of morphine, the % MPE of rats from chronic morphine treatment group (Mor group) was significantly decreased compared with the control group, indicating morphine tolerance model was successfully established (Figure 1A). Hargreaves test data also showed that morphine tolerance was established (Figure 1B). To investigate the possible link between miR-219-5p and morphine tolerance, we first analyzed the temporal changes in miR-219-5p expression of the spinal cord of rats using qRT-PCR. Compared with the control group, the data showed that miR-219-5p expression of Mor group declined on day 3 after morphine administration, to a minimum on day 7 (Figure 1C), correlating with the development of morphine tolerance (Figure 1A).

### Overexpression of miR-219-5p attenuates the development of morphine tolerance

**Figure 2:**
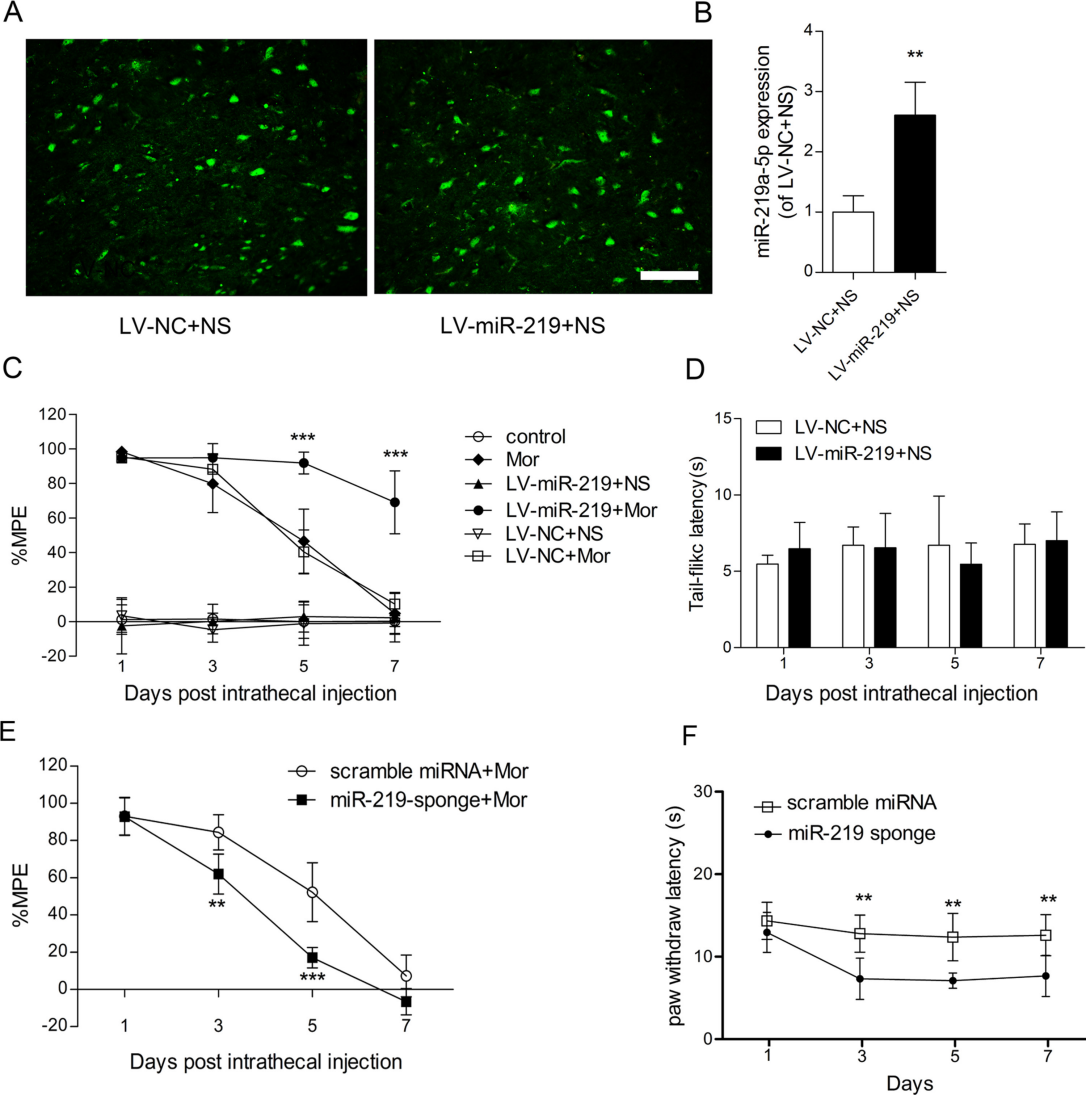
Overexpression of miR-219-5p attenuates the development of morphine tolerance **(A)** Green fluorescent protein (GFP) was expressed in the lumbar spinal cord (L3~L4) 10 days after injection of LV-miR-219 and LV-NC followed by 7 days of intrathecal saline injection. Scale bar = 200 μm. **(B)** The expression of miR-219-5p was upregulated on the day 10 after lentivirus injection. Rats were injected with LV-miR-219 and LV-NC, 3 days before normal saline infusion (*n* = 4, **P < 0.01, compared with LV-NC+NS, by Student's *t*-test). **(C)** Overexpression of miR-219-5p attenuated the development of morphine tolerance. (*n* = 6, ***P < 0.001, compared with LV-NC+Mor, by two-way ANOVA followed by Bonferroni correction); Mor = morphine (10 μg/10 μL, twice daily) intrathecal injection for 7 days; control = saline (10 μL, twice daily) intrathecal injection for 7 days; LV-miR-219+NS/Mor = intrathecal injection with LV-miR-219 3 days before consecutive normal saline/morphine infusion; LV-NC+NS/Mor = intrathecal injection with LV-NC 3 days before consecutive normal saline/morphine infusion. **(D)** Basal tail-flick latency remained unchanged after overexpression of miR-219-5p. Basal tail-flick latencies were recorded on days 1, 3, 5, and 7 after morphine or saline infusion (*n* = 6). **(E)** Effect of miR-219 sponge on the development of morphine tolerance. (*n* = 5, **P < 0.01, ***P < 0.001, compared with scramble miRNA + Mor group, using two-way ANOVA followed by Bonferroni correction); scramble miRNA/miR-219-sponge+Mor = intrathecal injection with scramble miRNA or miR-219-sponge for 3 consecutive days after morphine infusion. **(F)** Effect of miR-219 sponge on the paw thermal threshold of naive rats. (*n* = 5, **P < 0.01, compared with scramble miRNA group, using two-way ANOVA followed by Bonferroni correction); scramble miRNA/miR-219 sponge = intrathecal injection of scramble miRNA or miR-219 sponge daily for 3 consecutive days on naive rats. All the data were expressed as mean ± SD.

To examine the specific contribution of miR-219-5p to the development of morphine tolerance, a lentiviral vector-mediated miR-219-5p was intrathecally injected in rats. Green fluorescence was immunochemically detected in the spinal cord after injection of lentivirus, indicating lentivirus was successfully transfected (Figure 2A). Furthermore, qRT-PCR data showed upregulation in miR-219-5p expression in the spinal cord, on the day 10 post-transfection (Figure 2B). The results indicated successful induction overexpression of miR-219-5p. We evaluated the effect of miR-219-5p overexpression on the development of morphine tolerance, intrathecal injection of the lentiviral miR-219-5p (LV-miR-219) and the negative control (LV-NC) 3 days before consecutive saline or morphine injection in rats. Chronic morphine administration led to rapid and complete tolerance in rats treated with saline or LV-NC. However, in rats exposed to LV-miR-219, chronic morphine administration failed to induce morphine tolerance, and on day 7 after morphine administration, morphine still had antinociceptive effect, with a 70% MPE (Figure 2C). These results suggested that overexpression of miR-219-5p prevented and attenuated the development of morphine tolerance. Moreover, the basal latencies of tail-flick tests were not significantly different between LV-NC and LV-miR-219 group, indicating that overexpression of miR-219-5p did not have a direct analgesic effect (Figure 2D). We then investigated the effect of downregulation of miR-219 on morphine tolerance. We treated the rats with miR-219 sponge for 3 consecutive days to decrease the expression of miR-219-5p. We found that miR-219 sponge could attenuate the antinociceptive effect of morphine and produce thermal hyperalgesia in naive rats (Figure 2E, 2F). These data suggested miR-219-5p in the spinal cord contributes to the development of morphine tolerance.

### Overexpression of miR-219-5p decreased CaMKIIγ and NR1 expression in the PC12 cells

To further explore the role of miR-219-5p in morphine tolerance, we investigated the relevant target genes. It was reported that miR-219-5p targeted CaMKIIγ to regulate NMDA receptor 1 (NR1) function [18]. Furthermore, both CaMKII family and NR1 were key regulators of morphine tolerance. Thus, we focused on CaMKIIγ, a subtype of CaMKII family, for further study.

**Figure 3:**
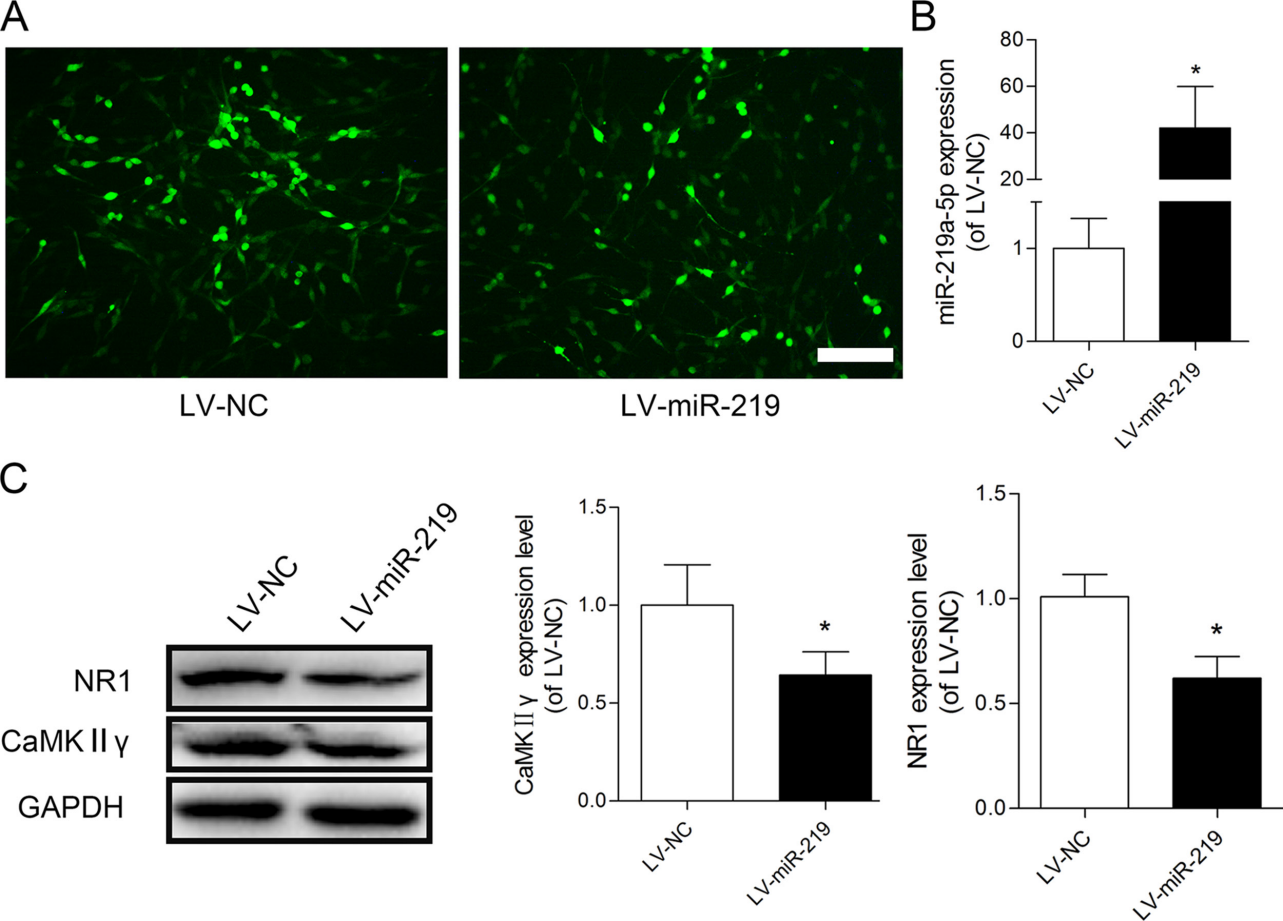
Overexpression of miR-219-5p decreased CaMKIIγ and NR1 expression in PC12 cells (**A**) GFP was visualized in PC12 cells after transfection with lentiviral miR-219-5p (LV-miR-219) and lentiviral negative control (LV-NC), Scale bar = 100 μm. (**B**) Expression of miR-219-5p was examined by qRT-PCR in PC12 cells, 5 days after lentivirus infection. LV-miR-219 induced robust upregulation of miR-219-5p expression in PC12 cell (*n* = 3, *P < 0.05, compared with LV-NC group, by Student's *t-test*). (**C**) Western blots showed the protein expression of CaMKIIγ and NR1 in PC12 cells after lentivirus infection. GAPDH was used as loading control. The expression of CaMKIIγ and NR1 was downregulated after PC12 cells were transfected with LV-miR-219 (*n* = 4, *P < 0.05, compared with LV-NC group, by Student's *t*-test). All the data were expressed as mean ± SD.

We investigated the effect of miR-219-5p overexpression on CaMKIIγ and NR1 by transfecting PC12 cells with LV-miR-219 and LV-NC. To confirm successful lentivirus delivery, cells were visualized microscopically to detect GFP fluorescence (Figure 3A). The qRT-PCR data showed that the expression of miR-219-5p was significantly increased in LV-miR-219-treated cells compared with LV-NC cells (Figure 3B). The Western blot data showed that LV-miR-219 treatment dramatically decreased the protein levels of both CaMKIIγ and NR1 (Figure 3C). These results were consistent with previous studies [18, 19], indicating that CaMKIIγ was the target of miR-219-5p and overexpression of miR-219-5p decreased CaMKIIγ and NR1 expression in the PC12 cells.

### miR-219-5p targets CaMKIIγ to alleviate morphine tolerance

**Figure 4:**
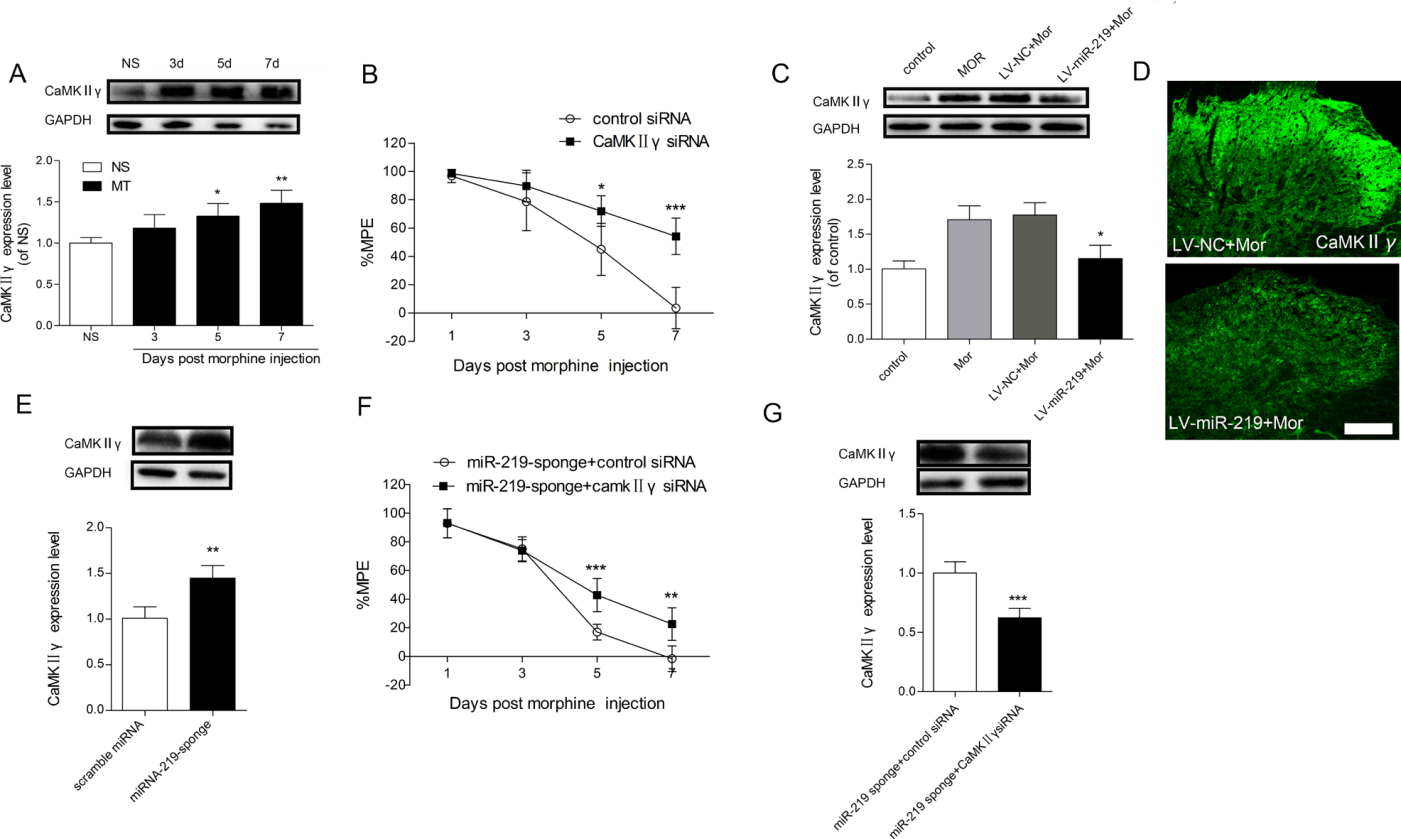
CaMKIIγ is responsible for miR-219-5p induced attenuation of morphine tolerance (**A**) Time course of CaMKIIγ changes in L4~L5 spinal cord during the development of morphine tolerance. Western blot of CaMKIIγ expression in the spinal cords of rats treated with saline infusion (NS) on the day 7, and in the morphine infusion group (MT) on days 3, 5, and 7. GAPDH was used as loading control (*n* = 3, *P < 0.05, compared with NS group, by one-way ANOVA followed by Bonferroni test). (**B**) CaMKIIγ siRNA attenuates the development of morphine tolerance. CaMKIIγ siRNA and control siRNA were intrathecally injected daily for 3 consecutive days after morphine infusion. (*n* = 5, *P < 0.05, ***P < 0.001, compared with control siRNA group, using two-way ANOVA followed by Bonferroni correction). (**C**) Expression of CaMKIIγ protein in the spinal cord 10 days after lentivirus injection. The increased expression of CaMKIIγ induced by chronic morphine treatment was reduced by overexpression of miR-219-5p (n = 3, *P < 0.05, compared with LV-miR-219+Mor, by one-way ANOVA followed by Bonferroni test). Control = saline (10 μL, twice daily) intrathecal injection for 7 days; Mor = Morphine (10 μg/10 μL, twice daily) intrathecal injection for 7 days; LV-miR-219/LV-NC+Mor = LV-miR-219 or LV-NC (10 μL) plus 7 days morphine infusion (10 μg/10 μL, twice daily). (**D**) Representative images of CaMKIIγ in the spinal cord by immunofluorescent labeling 10 days after lentivirus injection followed by consecutive morphine infusion. Scale bar = 100μm. (**E**) Expression of CaMKIIγ protein in the spinal cord of naive rats on the day 7 after scramble miRNA or miRNA-219-sponge intrathecal injection. (*n* = 4, **P < 0.01, compared with scramble miRNA group, by Student's *t*-test). (**F**) CaMKIIγ siRNA partially restore the loss of antinociceptive effect of morphine induced by miR-219 sponge. (*n* = 5, **P < 0.01, ***P < 0.001, compared with miR-219 sponge + control siRNA group, using two-way ANOVA followed by Bonferroni correction); miR-219 sponge + CaMKIIγ siRNA/control siRNA = miR-219 sponge was intrathecally injected for 3 days after consecutive morphine infusion, CaMKIIγ siRNA or control siRNA was intrathecally injected for 3 consecutive days from day 4 after morphine injection. (**G**) Expression of CaMKIIγ protein in the spinal cord on the day 7 after injection of miR-219 sponge and CaMKIIγ siRNA in morphine treated rats. (*n* = 4, ***P < 0.001, compared with miR-219 sponge+control siRNA group, by Student's *t*-test). All the data were expressed as mean ± SD.

To gain further insight into the mechanism of miR-219-5p in morphine tolerance, we investigated the expression of CaMKIIγ in the context of morphine tolerance. Western blot showed that CaMKIIγ was gradually increased after intrathecal injection of morphine (Figure 4A). And we found that knockdown of spinal CaMKIIγ by siRNA intrathecal injection restored the antinociceptive effect of morphine (Figure 4B), suggesting knockdown of CaMKIIγ could attenuate morphine tolerance. To further explore the interaction between miR-219-5p and CaMKIIγ in morphine tolerance, we first examined whether overexpression of miR-219-5p affected CaMKIIγ expression. Overexpression of miR-219-5p reversed the increased expression of CaMKIIγ by intrathecal injection of LV-miR-219 rather than LV-NC (Figure 4C). Immunochemistry results were consistent with Western blot results (Figure 4D). Then we investigated the effect of downregulation of miR-219-5p on the expression of CaMKIIγ, we found downregulation of miR-219-5p by miR-219 sponge increased the expression of CaMKIIγ (Figure 4E). Moreover, intrathecal injection of CaMKIIγ siRNA partially abolished miR-219 sponge induced decline of %MPE after morphine injection (Figure 4F, 4G). Therefore, these data suggested that overexpression of miR-219-5p alleviated morphine tolerance by targeting CaMKIIγ.

### NMDAR1 is involved in miR-219 mediated regulation of morphine tolerance

**Figure 5:**
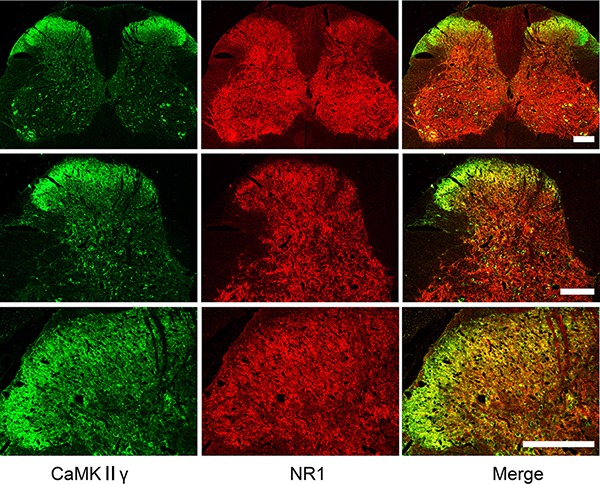
Localization of CaMKIIγ and NR1 in the spinal cord Double immunofluorescence labeling showing abundant expression of both CaMKIIγ (green) and NR1 (red), and co-expression of CaMKIIγ and NR1(yellow) in the spinal cord under different levels of magnification. Scale bar = 200 μm.

**Figure 6:**
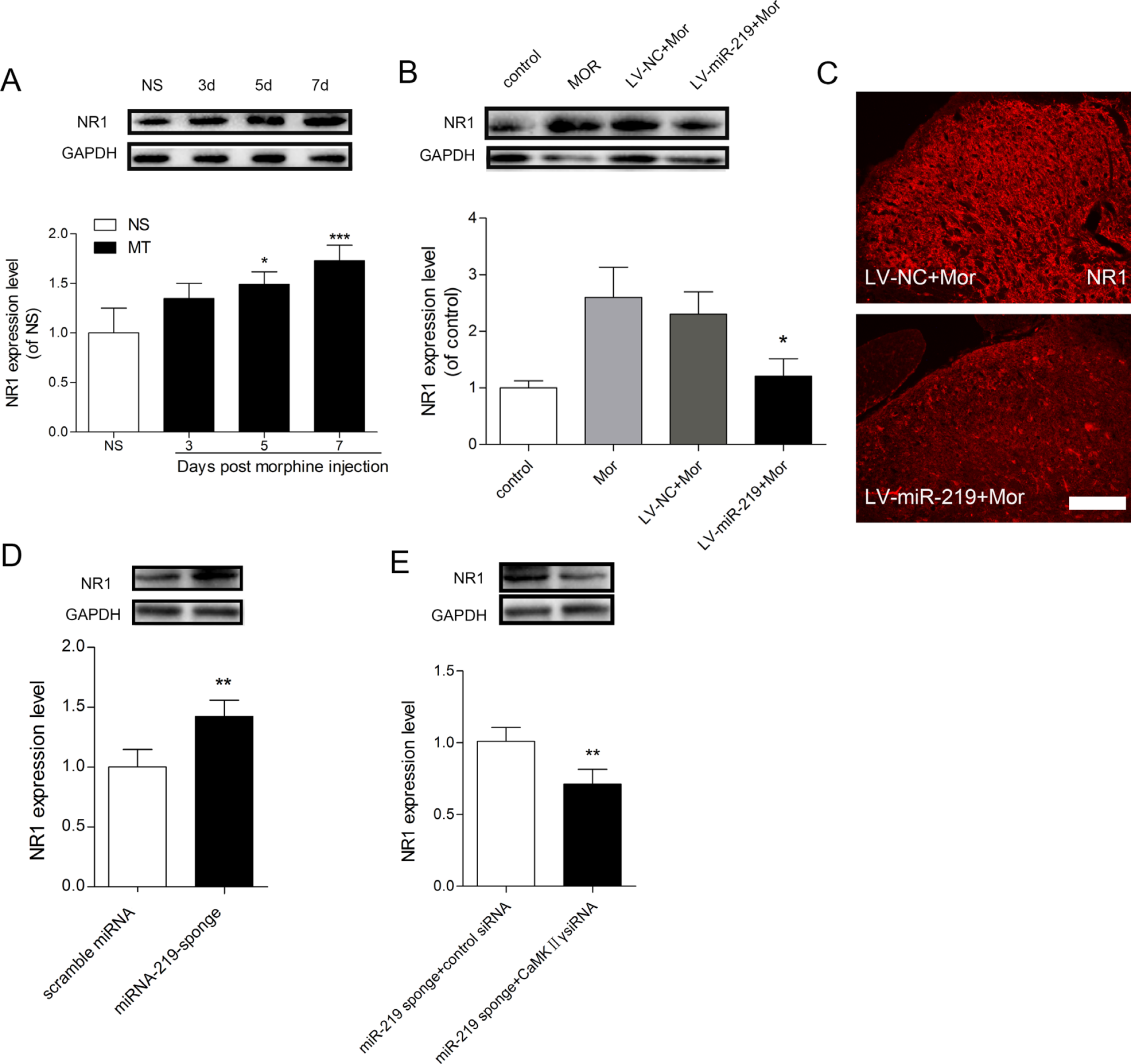
NR1 is involved in miR-219-5p mediated regulation of morphine tolerance (**A**) Western blot analysis of changes in NR1 expression of the spinal cord (L4~L5) during morphine tolerance. GAPDH was used as a loading control (*n* = 3, *P < 0.05, compared with NS group, by one-way ANOVA followed by Bonferroni test). (**B**) miR-219-5p overexpression reduced NR1 expression in the spinal cord after chronic morphine treatment (*n* = 3, *P < 0.05, compared with LV-miR-219 +Mor, by one-way ANOVA followed by Bonferroni test). (**C**) Representative image of NR1 expression in spinal cord 10 days after lentivirus injection Scale bar = 100 μm. (**D**) Expression of NR1 protein in the spinal cord of naive rats on the day 7 after scramble miRNA or miRNA-219-sponge injection. (*n* = 4, **P < 0.01, compared with scramble miRNA group, by Student's *t*-test). (**E**) Expression of NR1 protein in the spinal cord on the day 7 after injection of miR-219 sponge and CaMKIIγ siRNA in morphine treated rats. (*n* = 4, **P < 0.001, compared with miR-219 sponge + control siRNA group, by Student's *t*-test). All the data were expressed as mean ± SD.

Our study found that miR-219-5p targeted CaMKIIγ to regulate morphine tolerance. However the specific mechanism under this process was not clear. A recent study revealed that CaMKIIγ was a downstream target in the NMDA signaling pathway, which modulated NMDAR trafficking [18, 21]. Our *in vitro* results showed that overexpression of miR-219-5p repressed the expression of NR1 in PC12 cells (Figure 3C). Moreover, it has been demonstrated that blocking NMDA receptor function attenuates morphine tolerance [22, 23]. Thus, we hypothesized that miR-219-5p target CaMKIIγ to regulate NMDAR1 (NR1) function in morphine tolerance. To test the hypothesis, we first examined the localization of CaMKIIγ and NR1 in the spinal cord. Double immunofluorescence labeling revealed that NR1 was co-expressed with CaMKIIγ in the dorsal horn of spinal cord (Figure 5), indicating that CaMKIIγ may interact with NR1 spatially. We next investigated the expression changes in NR1 after chronic morphine injection. We found that NR1 expression was gradually increased after morphine injection (Figure 6A), which was consistent with the changes in CaMKIIγ (Figure 4A), and increased NR1 expression was downregulated by the overexpression of miR-219-5p accompanied with downregulation of CaMKIIγ (Figure 6B, 6C). Moreover, we found the expression of NR1 was also upregulated in rats receiving miR-219 sponge (Figure 6D), and knockdown of CaMKIIγ by siRNA could block this effect (Figure 6E). Together, these results suggested that NR1 is involved in miR-219 mediated regulation of morphine tolerance.

## DISCUSSION

miRNAs regulate multiple neurological mechanisms. Our data support the role of miR-219-5p in attenuating morphine tolerance. In the present study, we investigated the dynamic changes in miR-219-5p and CaMKIIγ using the morphine tolerance model. We found that consecutive intrathecal administration of morphine decreased the expression of miR-219-5p in the spinal cord, and increased the expression of CaMKIIγ. We also demonstrated that intrathecal administration of LV-miR-219 prevented the development of morphine tolerance and in turn decreased the expression of CaMKIIγ. Moreover, we found that NMDA receptor subunit NR1 was regulated by CaMKIIγ and involved in miR-219-5p mediated attenuation of morphine tolerance. Our findings indicate that miR-219-5p may represent a novel treatment for morphine tolerance.

miR-219-5p was previously recognized as a brain-specific miRNA that was only expressed in the brain [24]. It was found to mediate various neuronal processes, such as cell proliferation, differentiation and myelin maintenance [25, 26]. miR-219-5p is deregulated in neuronal dysfunction. Previous studies reported that miR-219 was highly upregulated in the brain of schizophrenia patients [27], and downregulated in subventricular zone and hippocampus of mice with amyotrophic lateral sclerosis (ALS) [28]. Recent studies suggest that miR-219 was also involved in the pathophysiology of Alzheimer's disease (AD) and epilepsy [21, 29].

Our study found that chronic administration of morphine significantly down-regulated the expression of miR-219-5p. This study firstly described the altered expression of spinal miR-219-5p in morphine tolerance. After intrathecal administration of lentivirus, we found that the overexpression of miR-219-5p significantly alleviated morphine tolerance. However, the anti-nociceptive effect of morphine was not fully restored by LV-miR-219, suggesting the possible role of other miRNAs or unknown mechanisms. Consistent with previous study, we also found blocking miR-219-5p induced thermal hyperalgesia, indicating its potential role in pain management [19].

miRNA mostly regulates the target gene expression negatively. There are some studies have reported that miR-219-5p directly targets CaMKIIγ [18, 19]. Therefore, CaMKIIγ, a target gene of miR-219-5p was selected for further study. CaMKIIγ is a component of CaMKII enzyme family. CaMKII activation depends on Ca^2+^/calmodulin, and is a multifunctional protein kinase highly expressed in CNS. Activation of CaMKII in CNS has been shown to play a crucial role in gene expression, memory processing, learning and neuroplasticity [30–32]. The specific role of CaMKII in morphine tolerance was inconsistent. Lou et al. [33] found that the subtype of CaMKII was expressed differentially following acute and chronic morphine treatment. Acute morphine treatment increased CaMKII activity in rat hippocampus, with little alteration in protein levels. Chronic morphine treatment down-regulated CaMKII activity, and increased the protein levels of β isoform of CaMKII, with little effect on α isoform. Fan et al. [34] reported that down-regulation or inhibition of CaMKII strongly attenuated morphine tolerance and dependence. Repeated morphine treatment increased the expression of both α and β isoforms as well as CaMKII activity via induction of morphine sensitization [35].

However, the effect of morphine tolerance on the expression of CaMKIIγ in the spinal cord is unclear. In our study, we found that CaMKIIγ expression in the spinal cord was gradually upregulated after chronic morphine treatment, which was negatively correlated with changes in miR-219-5p expression. After overexpression of miR-219-5p, the increased expression of CaMKIIγ induced by chronic morphine exposure was significantly downregulated with attenuation of morphine tolerance, indicating that targets CaMKIIγ to regulate morphine tolerance. Our *in vitro* test also demonstrated that overexpression of miR-219-5p inhibited CaMKIIγ expression.

CaMKIIγ is involved in NMDAR-mediated neuroplasticity and psychiatric dysfunction [18]. CaMKIIγ is an integral downstream target in NMDA-mediated Ca^2+^ signaling and negatively regulates the expression of NMDAR1 to alleviate CFA-induced chronic inflammatory pain [18, 19]. NMDA receptor, which belongs to glutamatergic receptor system, plays an important role in central sensitization and neuronal plasticity [36, 37]. NMDA receptor is comprised of NR1, NR2A-D and NR3 subunits. Activation of NMDA receptor has been reported to play a crucial role in the development of morphine tolerance [38]. Chronic morphine treatment altered NMDA receptor expression and up-regulated the expression of NR1 [39]. Inhibition of NMDA receptor activity by its noncompetitive antagonist MK801 or NR1 antisense oligonucleotide alleviated morphine tolerance [22, 40]. Our current study showed that chronic morphine treatment was associated with a time-dependent upregulation of NR1 subunit in the spinal cord, which was consistent with a previous report [41]. Furthermore, our study demonstrated that NR1 expression was correlated with the expression of miR-219-5p. The expression of NR1 was downregulated after injection of miR-219-5p, and upregulated after injection of miR-219 sponge. Because NR1 was not the target gene of miR-219-5p, the expression change of NR1 following miR-219 disturbance may result from the changes of CaMKIIγ.

Consistent with our findings, Hu et al. [42] found that miR-219 in dorsal root ganglion (DRG) contributed to morphine tolerance by targeting CaMKIIγ to regulate brain-derived neurotrophic factor (BDNF) expression. Growing evidences suggested BDNF is involved in spinal plasticity and central sensitization and NR1 activation is enhanced by BDNF released both in the spinal cord and DRG [43, 44]. BDNF is produced by microglia or neuron, and through presynaptic receptor signal transduction pathways to promote glutamate release, at the same time, BDNF acts on the AMPA and enhanced NMDA activity by postsynaptic receptor pathway, and participates in and promote LTP [45–47]. Thus we cannot deny the possibility that BDNF may act as a mediator in miR-219-CaMKIIγ-NR1 pathway in the study. Further study is still needed to clarify the specific role of BDNF in miR-219 mediated regulation of morphine tolerance in the spinal cord.

In addition to CaMKIIγ, several other target genes of miR-219-5p have been studied. It has been reported that miR-219-5p targets EGFR to inhibit glioma cell proliferation and migration [48]. miR-219-5p also targets oncogene Sall4 to suppress colon cancer proliferation and invasion [49]. Santa-Maria et al. [29] reported that dysregulation of microRNA-219 promotes neurodegeneration through post-transcriptional regulation of tau. However, none of these target genes are related to morphine tolerance. As miR-219-5p has hundreds of target genes, we cannot exclude the possibility that other target genes are involved in the context of morphine tolerance.

Based on our experiments, our findings can be summarized as follows: chronic morphine treatment downregulates expression of miR-219-5p, which upregulates the expression of CaMKIIγ. Upregulation of CaMKIIγ increases NMDA receptor expression and activity, resulting in morphine tolerance. Overexpression of miR-219-5p silences the translation of CaMKIIγ, and inhibits expression of NR1, resulting in alleviation of morphine tolerance. However, our study is associated with a few limitations. Neither miR-219-5p mimic nor lentiviral-mediated overexpression increased the expression of miRNA to an appropriate level for optimal function with minimal side effects. Further studies are still needed to elucidate the precise mechanisms and potential side effects of miR-219-5p involved in attenuating morphine tolerance, and investigate other target genes of miR-219-5p in the context of morphine tolerance.

In conclusion, our results show that miR-219-5p is involved in morphine tolerance by targeting CaMKIIγ and then affects NR1 expression. Increasing miR-219-5p expression level by intrathecal administration of lentivirus-mediated miR-219-5p attenuates morphine tolerance and decreases CaMKIIγ and NR1 expression. Our study expands our knowledge of the functional role of miR-219-5p and provides a novel and promising strategy for the treatment of morphine tolerance.

## MATERIALS AND METHODS

### Animals

Male Sprague-Dawley rats, each weighing 220 g to 250 g (Experimental Animal Center of Central South University) were housed in plastic cages under a 12-h light/12-h dark cycle. Food and water were provided ad libitum. All the procedures were consistent with the guidelines approved by the Administrative Committee of Experimental Animal Care and Use of Central South University. The study was compliant with the Ethical Guidelines of the International Association for the Study of Pain [50]. Efforts were made to minimize the number of animals and all the behavioral tests were performed by an observer blinded to animal treatment.

### Induction of morphine tolerance

Intrathecal catheter implantation was performed according to the methods of Yaksh [51]. Briefly, a PE-10 catheter was inserted through a cistemal incision and advanced caudally into the subarachnoid space of lumbar enlargement (L3~L4). After catheter implantation, rats were allowed to recover for 3 days, and any rat with paralysis or motor weakness was excluded from the experiment. To induce morphine tolerance, 10 μg morphine sulfate (in a volume of 10 μL) was delivered via an intrathecal catheter twice daily for 7 d. The control group was injected with 10 μL normal saline. Injections were followed by administration of 10 μL normal saline to flush the catheter [52]. Morphine analgesia was assessed on the test day using the tail-flick test both before and 30 min after morphine administration.

### Tail-flick test

The tail-flick test using radiant heat was performed to assess morphine analgesia among the different groups as previously described [53]. The intensity of the heat source was adjusted to ensure a basal latency of 4~6 sec, and a cut-off latency of 15 sec was set to minimize tissue damage. Tail-flick test was performed both before (baseline latency) and 30 min after morphine administration. The results were converted to the maximal possible anti-nociceptive effect (% MPE). The % MPE was calculated as follows: % MPE = [(post-drug latencies-baseline latencies)/(cutoff time-baseline latencies)] ×100.

### Hargreaves thermal withdrawal latency test

Thermal paw withdrawal latency test was performed by a Hargreaves apparatus (Plantar test, 7370; Ugo Basile, Comerio, Italy) as previously described [54, 55]. For this measurement, rats were placed on a glass platform within transparent plastic cylinders. After 15 min of acclimation, a heat beam was focused on the plantar surface of one hind paw. Withdrawal latency of the hind paw from the heat source was recorded as the response latency. A 25 sec cutoff was set to prevent tissue damage. Three measurements were made with an interval of 5 min.

### PC-12 cell culture

Differenced PC-12 cells were maintained in RPMI 1640 media (Invitrogen) containing 10% fetal bovine serum (FBS) (Gibco), 50 units/mL of penicillin and 50 μg/mL streptomycin in a humidified atmosphere of 5% CO_2_ at 37°C. The cells were passed every two days.

### Lentiviral system

Lentiviral vector-mediated miR-219-5p (LV-miR-219) was purchased from Genepharma. The titer of lentivirus was 2 × 10^9^ TU/ml. The sequence of miR-219-5p was designed as follows: TGATTGTCCAAACGCAATTCT and cloned into pGLV3/H1/GFP+Puro vector. Scrambled oligonucleotides (TTCTCCGAACGTGTCACGT) were used as negative control (LV-NC). PC-12 cells were transfected with lentivirus at 100 MOI for 24 h, for the *in vitro* test. *In vivo*, LV-miR-219 was intrathecally injected via catheter 3 days before the induction of morphine tolerance. The GFP fluorescence in the PC-12 cells and spinal cord was monitored to confirm successful transfection, using a fluorescent microscope (Leica, Germany).

### miRNA sponge and siRNA

miR-219 sponge, scramble miRNA, CaMKIIγ siRNA and control siRNA were purchased from Genepharma. The target sequence of miR-219 sponge was designed as following: AGAATTGCGTTTGGACAATCA. For naive rats, miR-219 sponge (20 μg, 4 μL) and scramble miRNA was intrathecal injected daily for 3 consecutive days, for rats receiving continuous morphine, they were intrathecal injected for 3 consecutive days just after morphine injection. CaMKIIγ siRNA (sense 5′-GGAUAUGCCGACUUCUGAATT-3′, antisense 5′-UUCAGAAGUCGGGCAUAUCCTT-3′) (40 μM, 4 μL) and control siRNA were also intrathecal injected for 3 consecutive days after morphine or miR-219 sponge injection.

### Quantitative real-time PCR (qRT-PCR)

Total RNA from the lumbar spinal cord (L4~L5) or PC-12 cell was isolated with TRIzol (Invitrogen) according to the manufacturer's recommendations. Following DNase digestion, RNA quantity and quality was determined by Nanodrop 2000 (Thermo Scientific). RNAs were reverse transcribed using TaqMan microRNA Reverse Transcription kit (Applied Biosystems) according to the manufacturer's protocol. The qRT-PCR was performed using specific primers for miR-219-5p (GSP: 5′GGTGA TTGTCCAAACGG3′ R: 5′CAGTGCGTGTCGTGGA3′) in an ABI prism 7900HT system (Applied Biosystems) with Fast Start Universal SYBR Green Master (Rox) (Roche). U6 (F: 5′GCTTCGGCAGCACATATACTAAAAT3′ R: 5′CGCTTCACGAATTTGCGTGTCAT3′) was used for normalization. The qRT-PCR conditions were: 95°C for 10 min, 40 cycles of 95°C for 10 sec, and 60°C for 60 sec. All the samples were run in duplicate. The relative expression of miRNA was determined using the 2^(−ddCt)^ calculations [56], and expressed as fold change of control sample.

### Immunochemistry

Rats were transcardially perfused with 4% paraformaldehyde. The spinal cord in the lumbar enlargement was resected and post-fixed for 2 h before transfer to 25% PBS-sucrose overnight. After dehydration, the spinal cord was embedded with Tissue-Tek O.C.T compound (Sakura) and 10-μm-thick frozen sections were obtained. For immunochemistry, sections were rinsed twice in PBS, permeabilized with 0.3% Triton X-100 and blocked with 10% normal donkey serum (Jackson ImmunoResearch) for 1 h. The sections were incubated with the primary antibodies: rabbit anti-GFP (1:200, Cell Signal Technology); rabbit anti-CaMKIIγ (1:200, Abcam) and rabbit anti-NR1 (1:200 Boster) at 4°C overnight. Next day, sections were washed with PBS three times and incubated with Alexa Fluor 488 or 594-conjugated donkey anti-rabbit IgG (Jackson ImmunoResearch) for 2 h. After rinsing 3 times in PBS, the sections were visualized and documented with a Leica Observer Microscope (Leica, Germany).

### Western blot analysis

Tissues (L4~L5 spinal cord) and PC-12 cell samples were homogenized in RIPA lysis buffer containing 1 mM phenylmethylsulfonyl fluoride. After centrifugation at 20,000×g for 15 min, the supernatant was collected. The protein concentration was determined using the BCA protein Assay Kit (Pierce). Western blot was performed with 30 μg of protein extracts and the proteins were separated using 10% SDS-PAGE at 80~120V for 90 min. After electrophoresis, proteins were transferred to polyvinylidene difluoride (PVDF) membranes (Merck Millipore) at 250 mA for 50 min. Membranes were blocked with 5% nonfat milk in TBS buffer containing 0.2% Tween 20 (TBST) at room temperature for 1h to avoid non-specific binding sites. The membranes were probed at 4°C overnight with the following primary antibodies: rabbit anti-CaMKIIγ (1:2000, Abcam); rabbit anti-NR1 (1:1000, Boster) and rabbit anti-GAPDH (1:5000, Abcam). The membranes were washed with TBST three times and incubated with the secondary antibody: peroxidase-conjugated goat anti-rabbit IgG, at room temperature for 1.5 h. After rinsing in TBST three times, the blots were visualized using the enhanced chemiluminescence plus system (Merck Millipore). Western blot data were digitized and analyzed using Image Lab 3.0 (Bio-Rad). The protein density of CaMKIIγ and NR1 was normalized against the density of GAPDH.

### Statistical analysis

Data are presented as mean ± standard deviation (SD). The statistical significance was evaluated using Student's *t*-test when comparing with two groups. Multiple groups were compared using one-way or two-way ANOVA followed by Bonferroni multiple comparison tests. All the analyses were performed using Graphpad Prism 5.0 software. *P* < 0.05 was considered statistically significant.
